# New Appointment

**Published:** 1850-05

**Authors:** 


*New Appointment.*—Dr. Landon C. Rives, of Cincinnati, Ohio, has been appointed to the Chair of Materia Medica in the Medical College of Ohio, made vacant by the decease of the late Prof. Harrison.

